# Clinical Courses of IKAROS and CTLA4 Deficiencies: A Systematic Literature Review and Retrospective Longitudinal Study

**DOI:** 10.3389/fimmu.2021.784901

**Published:** 2022-01-11

**Authors:** Akihiro Hoshino, Etsushi Toyofuku, Noriko Mitsuiki, Motoi Yamashita, Keisuke Okamoto, Michio Yamamoto, Kenji Kanda, Genki Yamato, Dai Keino, Yuri Yoshimoto-Suzuki, Junji Kamizono, Yasuhiro Onoe, Takuya Ichimura, Mika Nagao, Masaru Yoshimura, Koji Tsugawa, Toru Igarashi, Kanako Mitsui-Sekinaka, Yujin Sekinaka, Takehiko Doi, Takahiro Yasumi, Yozo Nakazawa, Masatoshi Takagi, Kohsuke Imai, Shigeaki Nonoyama, Tomohiro Morio, Sylvain Latour, Hirokazu Kanegane

**Affiliations:** ^1^ Department of Pediatrics and Developmental Biology, Graduate School of Medical and Dental Sciences, Tokyo Medical and Dental University, Tokyo, Japan; ^2^ Laboratory of Lymphocyte Activation and Susceptibility to EBV Infection, INSERM UMR 1163, Imagine Institute, Paris, France; ^3^ Graduate School of Medicine and Faculty of Medicine, The University of Tokyo, Tokyo, Japan; ^4^ Department of Pediatrics, Yokohama Municipal Citizen’s Hospital, Yokohama, Japan; ^5^ Department of Pediatrics, Hikone Municipal Hospital, Hikone, Japan; ^6^ Department of Hematology/Oncology, Gunma Children’s Medical Center, Shibukawa, Japan; ^7^ Division of Hematology/Oncology, Kanagawa Children’s Medical Center, Yokohama, Japan; ^8^ Department of Pediatrics, Center Hospital of the National Center for Global Health and Medicine, Tokyo, Japan; ^9^ Department of Pediatrics, Kitakyushu City Yahata Hospital, Kitakyushu, Japan; ^10^ Department of Pediatrics, Kitakyushu Municipal Medical Center, Kitakyushu, Japan; ^11^ Department of Pediatrics, Yamaguchi University Graduate School of Medicine, Ube, Japan; ^12^ Department of Pediatrics, Ohta Nishinouchi Hospital, Koriyama, Japan; ^13^ Department of Rheumatology, Endocrinology and Nephrology, Faculty of Medicine and Graduate School of Medicine, Hokkaido University, Sapporo, Japan; ^14^ Department of Pediatrics, Hirosaki University Graduate School of Medicine, Hirosaki, Japan; ^15^ Department of Pediatrics, Nippon Medical School, Tokyo, Japan; ^16^ Department of Pediatrics, National Defense Medical College, Tokorozawa, Japan; ^17^ Department of Pediatrics, Hiroshima University Graduate School of Biomedical and Health Science, Hiroshima, Japan; ^18^ Department of Pediatrics, Kyoto University Graduate School of Medicine, Kyoto, Japan; ^19^ Department of Pediatrics, Shinshu University School of Medicine, Matsumoto, Japan; ^20^ Department of Community Pediatrics, Perinatal and Maternal Medicine, Graduate School of Medical and Dental Sciences, Tokyo Medical and Dental University, Tokyo, Japan; ^21^ Université de Paris, Paris, France; ^22^ Department of Child Health and Development, Graduate School of Medical and Dental Sciences, Tokyo Medical and Dental University, Tokyo, Japan

**Keywords:** IKAROS deficiency, CTLA4 deficiency, systematic literature review, retrospective longitudinal study, clinical course

## Abstract

IKAROS and CTLA4 deficiencies are inborn errors of immunity and show similar clinical phenotypes, including hypogammaglobulinemia and autoimmune diseases (ADs). However, the differences in clinical features and pathogenesis of these are not fully understood. Therefore, we performed systematic literature reviews for IKAROS and CTLA4 deficiencies. The reviews suggested that patients with IKAROS deficiency develop AD earlier than hypogammaglobulinemia. However, no study assessed the detailed changes in clinical manifestations over time; this was likely due to the cross-sectional nature of the studies. Therefore, we conducted a retrospective longitudinal study on IKAROS and CTLA4 deficiencies in our cohort to evaluate the clinical course over time. In patients with IKAROS deficiency, AD and hypogammaglobulinemia often develop in that order, and AD often resolves before the onset of hypogammaglobulinemia; these observations were not found in patients with CTLA4 deficiency. Understanding this difference in the clinical course helps in the clinical management of both. Furthermore, our results suggest B- and T-cell-mediated ADs in patients with IKAROS and CTLA4 deficiencies, respectively.

## Introduction

Common variable immunodeficiency (CVID) is the most prevalent disease of inborn errors of immunity (IEI) and is characterized by hypogammaglobulinemia with poor specific antibody production. In addition to increased susceptibility to recurrent bacterial infections, clinical manifestations of CVID include noninfectious complications, such as autoimmune diseases (ADs), enteropathy, lymphoproliferation/granulomatous diseases, and malignancy (1). Although CVID is clinically and genetically heterogeneous, identifying underlying genetic defects with the help of latest advances in sequencing technology can help classify CVID into homogeneous subgroups. Indeed, several next-generation sequencing studies have identified that 15%-30% of patients with CVID have candidate variants in genes including *NFKB1*, *NFKB2*, *CTLA4*, *LRBA*, *IKZF1*, *STAT3*, and *PIK3CD* (2).

Identifying genetic defects can help understand the pathogenesis of hypogammaglobulinemia or noninfectious complications. For example, IKAROS encoded by *IKZF1* is a key transcription factor of hematopoietic development, particularly early B-cell development. Patients with IKAROS haploinsufficiency (HI) or haploinsufficiency of the dimerization domain (DD) present with progressive B-cell deficiency, whereas IKAROS dominant-negative (DN) results in more severe manifestations involving B-, T-, and myeloid cell defects (collectively termed as IKAROS deficiency) (3–6). IKAROS also plays a role in tumor suppression. Somatic variants have been recurrently observed in B-cell precursor acute lymphoblastic leukemia (BCP-ALL), and patients with germline variants can develop BCP-ALL (3). Additionally, CTLA4 is an essential negative regulator of T-cell immune responses, and is expressed on regulatory (Treg) and activated effector T cells. CTLA4 HI (termed as CTLA4 deficiency) results in a hyperactivated immune system with T-cell infiltration in organs, autoimmunity, or both (7, 8). Although not completely clear, bone marrow infiltration of T cells or impaired germinal center formation may lead to hypogammaglobulinemia with reduced number of memory B cells (9). Considering this condition, the clinical use of abatacept, a CTLA4 fusion protein, is a reasonable therapeutic option.

Subdividing CVID by identifying its genetic defects has reduced the sample size of the studies. Early studies on CVID, especially cross-sectional studies, that clarified the clinical manifestations and appropriate management practices used large heterogeneous cohorts of 100 to >1,000 patients (10). However, recent studies on a monogenic disease included smaller cohorts of 10-100 patients (3, 4). Although studies on homogeneous cohorts could more accurately characterize the clinical features of the diseases, errors and biases may occur due to the small size. Therefore, world surveys or systematic literature reviews have been conducted to increase the sample size (11, 12). However, such studies are associated with potential problems as described below, thus warranting careful evaluations. Moreover, longitudinal studies are more suitable for studying a small number of patients rather than a large cohort due to the efficiency of time and cost (**Supplemental Table 1**) (13).

The primary aim of this study was to clarify the limitations of performing a literature review on IKAROS and CTLA4 deficiencies and to emphasize that a longitudinal study could compensate for its limitations. The secondary aim was to provide a possible pathogenesis for complications through a longitudinal study. This study focused on IKAROS and CTLA4 deficiencies from among the many known genetic defects because cohorts for these conditions have been previously described (4, 11) and the pathogenesis of at least one complication is well understood. Here, we performed systematic literature reviews for IKAROS and CTLA4 deficiencies and highlighted the associated problems, including case-publication bias, lack of data, and lack of information about the clinical course over time. This is most likely due to the presence of multicenter and cross-sectional studies. Furthermore, we conducted a retrospective longitudinal study on IKAROS and CTLA4 deficiencies in our Japanese cohort and determined a different onset order of hypogammaglobulinemia and AD in the two conditions. Therefore, this study aimed to emphasize that longitudinal studies can help understand the clinical presentation of diseases, such as IEI, with a small sample size and may provide a further understanding of the pathogenesis and aid in better clinical management.

## Materials and Methods

### Systematic Literature Review

We searched PubMed and Web of Science databases for articles published in English from April 2012 to June 2021 on IKAROS deficiency and from September 2014 to June 2021 on CTLA4 deficiency. The search terms were a “IKAROS haploinsufficiency”, “IKAROS haploinsufficient”, “IKAROS deficiency”, “IKAROS deficient”, “IKAROS mutation”, “IKAROS mutations”, “IKAROS variant”, “IKAROS variants”, “IKZF1 mutation”, “IKZF1 mutations”, “IKZF1 variant”, and “IKZF1 variants” for IKAROS deficiency, and “cytotoxic T-lymphocyte-associated protein 4 haploinsufficiency”, “cytotoxic T-lymphocyte-associated protein 4 haploinsufficient”, “cytotoxic T-lymphocyte-associated protein 4 deficiency”, “cytotoxic T-lymphocyte-associated protein 4 deficient”, “cytotoxic T-lymphocyte-associated protein 4 insufficiency”, “cytotoxic T-lymphocyte-associated protein 4 insufficient”, “CTLA4 haploinsufficiency”, “CTLA4 haploinsufficient”, “CTLA4 deficiency”, “CTLA4 deficient”, “CTLA4 insufficiency”, “CTLA4 insufficient”, “CTLA-4 haploinsufficiency”, “CTLA-4 haploinsufficient”, “CTLA-4 deficiency”, “CTLA-4 deficient”, “CTLA-4 insufficiency”, “CTLA-4 insufficient”, “CTLA4 mutation”, “CTLA4 mutations”, “CTLA4 variant”, and “CTLA4 variants” for CTLA4 deficiency. A search was performed using the Preferred Reporting Items for Systematic Reviews and Meta-Analyses guidelines for article identification, screening, eligibility, and inclusion (**Supplemental Figure 1**). Patients with insufficient details on clinical manifestations and with not functionally tested variants were excluded. However, those with nonsense or frameshift variants were included even if untested. IKAROS DN was evaluated separately as such patients have distinct clinical features.

### Retrospective Longitudinal Study

We identified 16 patients with IKAROS deficiency and 31 with CTLA4 deficiency in our registry of IEI referred to the Tokyo Medical and Dental University. One patient with IKAROS DN was excluded. Data on clinical presentation with its course over time, serum immunoglobulin levels, and treatment were retrospectively surveyed by reviewing medical records or questionnaires sent to physicians. Patients 1.1-20.1 and 22.1 have been previously reported (4, 5, 9, 11, 14–20). AD onset was calculated as the number of AD onset per 1 person-years, which is the sum of the patient follow-up duration (years). Incidence of AD remission was calculated as the number of AD remission per 1 person-years, which is the sum of the patient AD duration (years).

### Data Evaluation

Enteropathy, granulomatous and lymphocytic interstitial lung disease (GLILD), central nervous system (CNS) involvement, and other symptomatic lymphoproliferation/granulomatous diseases were included in AD as they are inflammatory complications and most likely associated with impaired immunological tolerance and lymphocyte infiltration (11). Asymptomatic lymphoproliferation, including lymphadenopathy and splenomegaly, was excluded from AD. The onset of hypogammaglobulinemia was defined as the onset of susceptibility to infection if serum immunoglobulin levels were not tested. Additionally, IgA and specific antibody deficiencies were excluded from hypogammaglobulinemia to emphasize the role of severe B-cell development/differentiation arrest.

In the longitudinal study, AD remission was defined as absent or minimal symptoms, normal laboratory data without treatment, and no relapse until the last follow-up. For patients who underwent hematopoietic stem cell transplantation (HSCT), onset and remission were evaluated for the period before HSCT. Furthermore, if hypogammaglobulinemia and AD simultaneously identified, AD onset was considered to be before that of hypogammaglobulinemia, because serum IgG levels and B-cell counts were further decreased in all available patients.

### Statistical Analyses

For statistical analyses and construction of Kaplan-Meier curves, GraphPad Prism 8 software (GraphPad) was used. The groups were compared using Mann-Whitney *U*-test or Wilcoxon signed-rank test for numerical data and chi-square test for non-numerical data. Moreover, 95% confidence intervals (CIs) were calculated using the exact binomial method (Clopper-Pearson method). Differences were considered significant at *P* values of <0.05. For constructing Kaplan-Meier curves, we defined censoring as loss of follow-up or HSCT.

### Functional Assay

Electrophoresis mobility shift assay (EMSA) and co-immunoprecipitation (Co-IP) assay were performed as previously described (4). For EMSA, HEK293T cells were transfected with pCMV3-HA-IKAROS (wild-type [WT] or mutants). Then, the nuclear protein was extracted and incubated with DY682 infrared dye-labeled double-strand IK-bs4 probe (forward: 5’-TGACAGGGAATACACATTCCCAAAAGC-3’; reverse: 5’-GCTTTTGGGAATGTGTATTCCCTGTCA-3’). DNA-protein complexes were separated using acrylamide gels and analyzed using an Odyssey CLx infrared scanner (Li-Cor). For Co-IP, HEK293T cells were co-transfected with pFLAG-CMV2-IKAROS (WT) and pCMV3-HA-IKAROS (WT or mutants). Then, cell lysates were immunoprecipitated with Protein G-Sepharose 4 Fast Flow (GE Healthcare) and mouse anti-FLAG antibody (F3165, Sigma), eluted, and separated using standard sodium dodecyl sulftate-polyacrylamide gel electrophoresis (SDS-PAGE). Finally, western blotting was performed using the rabbit anti-HA antibody (H6908, Sigma).

Intracellular CTLA4 expression was evaluated using flow cytometry. Peripheral blood mononuclear cells stimulated with anti-CD3/CD28 beads were stained with anti-CD4-PC7 (SFCI12T4D11 (T4), Beckman Coulter), fixed, permeabilized, and stained for anti-CTLA4-PE-Cy5 (BNI3, BD Biosciences) and anti-FOXP3-Alexa Fluor 647 (236A/E7, eBioscience).

## Results

### Systematic Literature Reviews

First, systematic literature reviews of IKAROS and CTLA4 deficiencies were performed, and 122 and 177 articles, respectively, were identified. Of those, 19 and 28 articles were considered eligible for systematic review. Furthermore, 95 and 246 patients were reported in these articles, and, after excluding patients to both groups, 90 and 179 unique patients remained for data analysis, respectively (**Supplemental Figure 1** and **Supplemental Table 2**). Previous reviews and world surveys have described genetic and immunological analyses, complication types, treatment, and outcomes (9, 11, 12, 21). Therefore, we primarily focused on complications and their clinical course.

### IKAROS Deficiency

**Figure 1:**
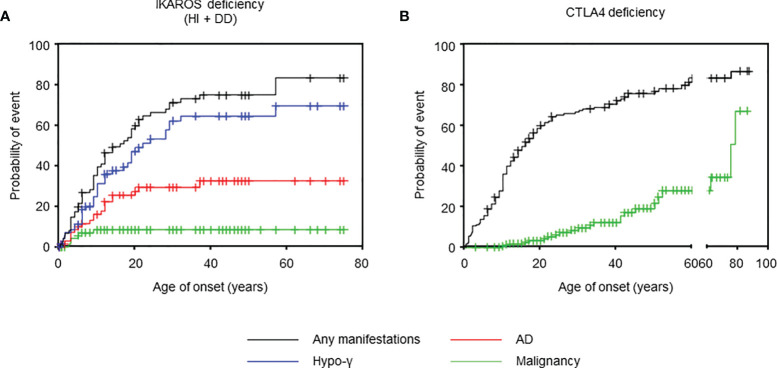
Age of onset in IKAROS and CTLA4 deficiencies. **(A)** Cumulative incidence of any manifestations (n = 77), hypogammaglobulinemia (n = 73), autoimmune disease (n = 72), and malignancy (n = 75) in IKAROS HI and IKAROS DD. **(B)** Cumulative incidence of any manifestation (n = 158) and malignancy (n = 166) in CTLA4 deficiency. AD, autoimmune disease; hypo-γ, hypogammaglobulinemia.

**Figure 2:**
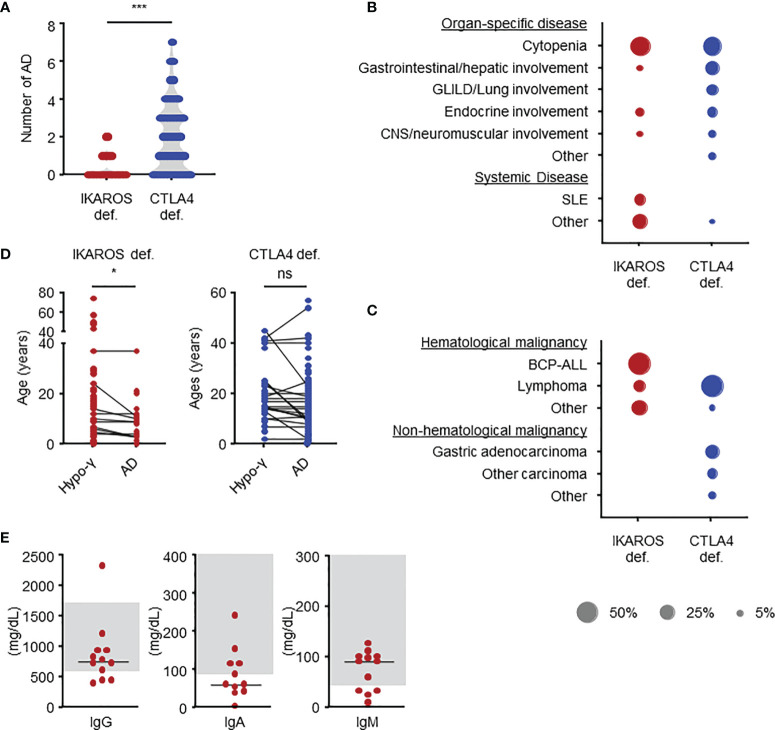
Clinical manifestations in IKAROS and CTLA4 deficiencies as determined by systemic literature reviews. **(A)** Number of autoimmune diseases. **(B)** Types of autoimmune diseases in terms of percentage of total number. **(C)** Types of malignancies. **(D)** Comparison of age at the onset. **(E)** Serum immunoglobulin levels at the onset of autoimmune disease in patients with IKAROS deficiency. The groups were compared using Mann-Whitney *U*-test in **(A)** and Wilcoxon signed-rank test **(D)**. *P < 0.05, ***P < 0.001. AD, autoimmune disease; BCP-ALL, B-cell precursor acute lymphoblastic leukemia; hypo-γ, hypogammaglobulinemia; ns, not significant; SLE, systemic lupus erythematosus.

This systematic review evaluated 90 patients with IKAROS deficiency bearing 28 distinct *IKZF1* variants: 66 patients (20 variants) with IKAROS HI, including large deletion and early truncation; 16 patients (6 variants) with IKAROS DD; and 8 patients (two variants) with IKAROS DN (**Table 1**). Data for IKAROS HI and DD were combined and evaluated as the number of patients was small and a similar tendency was observed in both groups (**Table 1**, **Supplemental Figure 2** and **Supplemental Table 3**). However, patients with IKAROS DN were separately evaluated as they had a phenotype distinct from CVID. In patients with IKAROS HI and DD, the median age and interquartile range at the last follow-up were 24 (13-45) years. Of 82 patients, 55 (67.1%) were symptomatic, and the median age at onset was 9 (4-19) years. Kaplan-Meier curves were constructed for 73, 72, and 7 5patients, including asymptomatic patients, whose ages at the onset of hypogammaglobulinemia, AD, and malignancy were available (**Figure 1A**). The cumulative incidence was 44.5%, 26.1%, and 9.7% at 20 years, and 61.3%, 31.1%, and 9.7% at 40 years, respectively. Although hypogammaglobulinemia developed even after 50 years of age, the cumulative incidence curves of AD and malignancy almost peaked within the first 20 years of life. These observations differ from previous reports on increased noninfectious complications with age in patients with CVID (22). Of the 23 patients with AD, 18 (78.3%) had only one AD (**Figure 2A**). Autoimmune cytopenia and systemic AD, such as systemic lupus erythematosus (SLE) and antiphospholipid syndrome (APS), were the most common (**Figure 2B**). BCP-ALL was the most common malignancy (**Figure 2C**). When each patient was separately evaluated, AD developed earlier or simultaneously as hypogammaglobulinemia (**Figure 2D**). Indeed, of 12 ADs in 10 patients available, only three showed hypogammaglobulinemia at the onset and none had agammaglobulinemia (**Figure 2E**). Furthermore, autoantibodies were detected in many patients (**Supplemental Table 4**).

**Table 1:** Baseline description of individuals with IKAROS deficiency (HI and DD) and CTLA4 deficiency in the systematic literature reviews.

	IKAROS HI and DD	CTLA4 def.	*P*-value
Number of patients	82	179	
Sex (M/F)	42/40 (n = 82)	91/88 (n = 179)	0.954
Age at last follow-up	24 [13-45] (n = 76)	26 [18-46] (n = 167)	0.108
Age at onset	9 [4-19] (n = 55)	11 [6-18] (n = 119)	0.203
Age at onset of hypo-γ	10 [6-19] (n = 39)	17 [12-25] (n = 33)	**0.010**
Age at onset of AD	9 [3-14] (n = 20)	10 [6-17] (n = 101)	0.301
Age at onset of malignancy	4 [3-6] (n = 6)	33 [21-50] (n = 23)	**<0.001**

The median ages are shown [with 25th and 75th percentiles] (year).

Bold numbers indicate the statistically significant correlations.

AD, autoimmune disease; hypo-γ, hypogammaglobulinemia.

In patients with IKAROS DN, the median ages at the last follow-up and onset were 10 (3-19) and 0.6 (0.3-1) years, respectively. All developed infections, including Pneumocystis pneumonia, in infancy. One patient developed T-cell ALL at the age of 13 years, and AD was not observed.

### CTLA4 Deficiency

We also evaluated 179 patients with CTLA4 deficiency bearing 61 distinct *CTLA4* variants (**Table 1**). The median age at the last follow-up was 26 (18-46) years. At onset, the median age in 133 (74.3%) symptomatic patients was 11 (6-18) years. The cumulative incidence of any manifestations was 60.2% at 20 years old and 72.4% at 40 years old, whereas that of malignancy was 3.4% at 20 years old and 12.3% at 40 years old (**Figure 1B**). However, because the ages at hypogammaglobulinemia and AD onset were unavailable in 25 (14.0%) and 38 (21.2%) patients, respectively, the cumulative incidence of each complication could not be evaluated. In available patients, hypogammaglobulinemia and malignancy developed at a significantly higher age than those with IKAROS deficiency (**Table 1** and **Supplemental Figure 3**). In contrast, 93/128 (72.7%) patients had two or more ADs without the tendency to develop earlier than hypogammaglobulinemia (**Figures 2A, D**). Although various organ-specific ADs were observed, systemic AD was rare (**Figure 2B**). Furthermore, lymphoma and gastric adenocarcinoma were the most common malignancy (**Figure 2C**).

### Limitations of Systematic Literature Reviews

**Figure 3:**
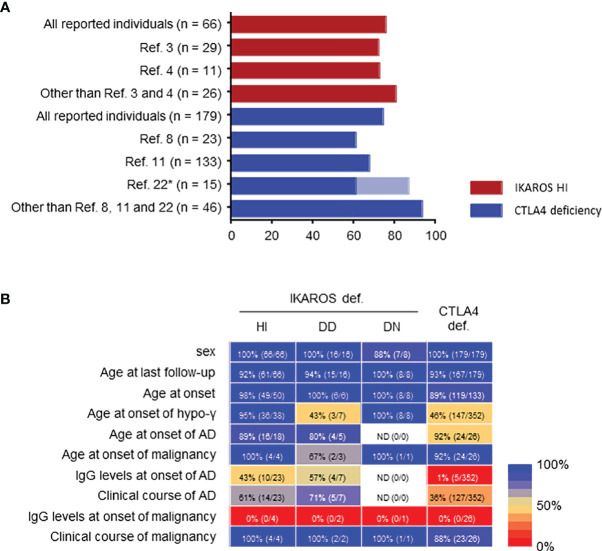
Limitations of systemic literature reviews. **(A)** Penetrance in IKAROS HI and CTLA4 deficiencies. *Penetrance was described as 86.7% (13/15) in ref. 22, but re-evaluated as 60% (9/15) in ref.^11^ in the same patients. **(B)** Percentage of clinical data available presented as a heatmap: blue, higher percentage available; red, lower percentage available. If even a few data were described, it was considered as available. AD, autoimmune disease; hypo-γ, hypogammaglobulinemia.

A major limitation of these systematic literature reviews is potential case-publication bias. For example, only two articles on IKAROS deficiency and three on CTLA4 deficiency (some patients were overlapped) described the clinical manifestations of substantial cohorts of >10 patients (3, 4, 8, 11, 23). The penetrance was 72.4%-72.7% for IKAROS HI and 67.7%-86.7% for CTLA4 deficiency in these cohorts. However, except for those five articles, the total patients showed a penetrance of 80.8% and 93.5%, respectively, suggesting an enrichment of symptomatic and likely severe or atypical cases when reported as single, few patients or family case reports (**Figure 3A**). Furthermore, in addition to the lack of clinical or laboratory data, there was little information about the clinical course over time, including the remission of complications, another major limitation due to the multicenter and cross-sectional nature of the studies (**Figure 3B**).

## Retrospective Longitudinal Study Involving Our Cohort

These systematic literature reviews suggest that patients with IKAROS deficiency develop in the order of AD and hypogammaglobulinemia, whereas those with CTLA4 deficiency do not. Therefore, to evaluate the clinical course over time in detail, we conducted a retrospective longitudinal study on IKAROS and CTLA4 deficiencies in our Japanese cohort.

### Patient Cohort and Functional Assays

Our cohort identified 16 patients with *IKZF1* variants bearing 10 distinct variants and 31 patients with *CTLA4* variants bearing 16 distinct variants (**Supplemental Table 5**). First, we functionally tested three previously untested *IKZF1* variants, K157del, F490del, and H508W and three novel CTLA4 variants, V84A, C129R, and P162fs. Three *IKZF1* variants were located on the DNA-binding or dimerization domain and are predicted to affect the maintenance of zinc finger structure or the coordination of zinc atom (**Supplemental Figure 4A**). The consequences of *IKZF1* variants were examined with mutant proteins transiently expressed in HEK293T cells. Furthermore, EMSA showed that the K157del mutant protein did not bind the IKAROS consensus sequence without a DN effect, indicating a HI variant (**Supplemental Figure 4B**). Co-IP assay showed that mutants F490del and H508W failed to bind to WT IKAROS, indicating DD variants (**Supplemental Figure 4C**). Additionally, CTLA4 protein expression was measured in FOXP3^+^ T cells from patients with *CTLA4* V84A, C129R, and P162fs variants. All three variants resulted in reduced CTLA4 expression (**Supplemental Figure 4D**). As IKAROS DN shows distinct clinical features, 15 patients with IKAROS deficiency (13 with HI and two with DD), excluding one with IKAROS DN, and 31 with CTLA4 deficiency were evaluated.

### Case Reports

**Figure 4:**
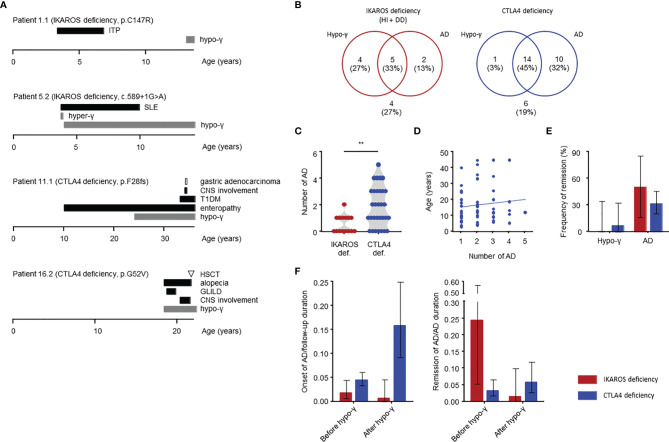
Clinical manifestations in IKAROS and CTLA4 deficiencies determined by longitudinal study. **(A)** Case reports of four patients. The AD duration is defined as from onset to the end of treatment with no symptoms or laboratory abnormalities. CNS, central nervous system; GLILD, granulomatous, and lymphocytic interstitial lung disease; HSCT, hematopoietic stem cell transplantation; hyper-γ, hypergammaglobulinemia; SLE, systemic lupus erythematosus; T1DM, type 1 diabetes mellitus. **(B)** Distribution of complications. **(C)** Number of autoimmune diseases. **(D)** Total number of autoimmune disease and age at onset for CTLA4 deficiency. **(E)** Frequency of remission of hypogammaglobulinemia and autoimmune disease. **(F)** Incidence of autoimmune disease onset or remission before and after onset of hypogammaglobulinemia shown as per 1 person-year. The person-year is the sum of the patient follow-up deration (years) for the onset, and the sum of the patient autoimmune disease duration (years) for the remission. The groups were compared using a Mann-Whitney *U*-test in **(C)**. **P < 0.01. Thin bars are presented as 95% CIs in **(E, F)**. AD, autoimmune disease; hypo-γ, hypogammaglobulinemia.

Herein, we describe four patients who had typical clinical courses (**Figure 4A**). Patient 1.1 with IKAROS HI is a 13-year-old boy presenting with immune thrombocytopenia (ITP) at the age of 3 years with increased platelet-associated IgG. Low serum IgA and IgM levels were observed at ITP diagnosis (IgG, 784 mg/dL; IgA, 2 mg/dL; and IgM, 9 mg/dL). His platelet counts were between 50,000 and 100,000/μL but normalized at age 6. His serum IgG levels decreased to <500 mg/dL since the age of 13 years.

Patient 5.2 is a 14-year-old boy with IKAROS HI. He developed SLE at age 3 years. At SLE diagnosis, hypergammaglobulinemia was present (IgG, 2,329 mg/dL; IgA, 154 mg/dL; and IgM, 97 mg/dL) with several positive autoantibodies, including antinuclear, antidouble-stranded DNA, anticardiolipin and antiribonucleoprotein antibodies. Disease activity in this patient was severe, and he required methylprednisolone pulse and immunoadsorption; however, he subsequently received prednisolone for 7 years without showing SLE-related symptoms. Hypogammaglobulinemia was detected at the same time as the SLE symptoms disappeared. He has been in clinical remission for 3 years without SLE treatment.

Patient 11.1 initially presented with enteropathy at the age of 10 years. He had hypogammaglobulinemia and started immunoglobulin replacement therapy when he was 24 years old. He had enteropathy and developed additional complications of type 1 diabetes and CNS involvement in his 30s. Additionally, he developed gastric adenocarcinoma at 34 years, which led to his death from sepsis 2 years after gastrectomy.

Patient 16.2 presented with pneumonia and hypogammaglobulinemia at the age of 18 years, he also had alopecia. He developed GLILD and showed CNS involvement after 2 years. Because those complications were refractory to glucocorticoids, immunosuppressant agents, and abatacept, he received HSCT at 21 years of age. Unfortunately, he died of HSCT-related complications 11 months after HSCT.

### Clinical Manifestations and Course

Patient characteristics are presented in **Table 2**. In our cohort, four patients with CTLA4 deficiency underwent HSCT. These patients were evaluated before undergoing HSCT as the procedure could replace the immune system. The median age at last follow-up was 18 (15-41) years for patients with IKAROS deficiency and 29 (20-47) years for those with CTLA4 deficiency. Hypogammaglobulinemia and AD were identified in 9 (60%) and 7 (47%) patients with IKAROS deficiency, and 15 (48%) and 24 (77%) patients with CTLA4 deficiency (**Figure 4B**). More than half of these patients developed both complications. Consistent with the results of the systematic literature reviews, the number of ADs per patients was higher in CTLA4 deficiency (**Figures 4C, D**).

**Table 2:** Baseline description of individuals with IKAROS deficiency (HI and DD) and CTLA4 deficiency in the longitudinal study.

	IKAROS HI and DD	CTLA4 def.	*P*-value
Number of patients	15	31	
Sex (M/F)	8/7 (n = 15)	14/17 (n = 31)	0.603
Age at last follow-up	18 [15-41] (n = 15)	29 [20-47] (n = 31)	0.160
Age at onset	10 [4-11] (n = 12)	10 [9-20] (n = 25)	0.060
Age at onset of hypo-γ	10 [9-12] (n = 9)	24 [14-35] (n = 15)	**0.005**
Age at onset of AD	5 [4-18] (n = 7)	10 [9-19] (n = 24)	0.310
Age at onset of malignancy	(n = 0)	31 (n = 2)	

The median ages are shown [with 25th and 75th percentiles] (year).

Bold number indicates the statistically significant correlation.

AD, autoimmune disease; hypo-γ, hypogammaglobulinemia.

Among the 9 and 15 patients with hypogammaglobulinemia in IKAROS and CTLA4 deficiency conditions, respectively, only one patient with CTLA4 deficiency (patient 15.1) achieved remission (**Figure 4E**). Patient 15.1 had mild hypogammaglobulinemia (IgG, 456 mg/dL; IgA, 95 mg/dL; and IgM, 35 mg/dL) but it improved after starting abatacept treatment. Moreover, 4 (50%) of 8 patients with ADs and 18 (32%) of 57 with ADs achieved remission with IKAROS and CTLA4 deficiencies, respectively (**Figure 4E**).

The number of patients with AD onset and remission was compared between before and after hypogammaglobulinemia onset using person-year method. The onset of AD per 1 person-year, which is the sum of the patient follow-up duration, was highest in CTLA4 deficiency after the onset of hypogammaglobulinemia. However, it was higher before the onset of hypogammaglobulinemia in IKAROS deficiency (**Figure 4F**). Alternatively, the number of remission of AD per 1 person-year, which is the sum of the patient AD duration, was highest in IKAROS deficiency before the onset of hypogammaglobulinemia, whereas remission was achieved in a few patients with CTLA4 deficiency (**Figure 4F**).

## Discussion

**Figure 5:**
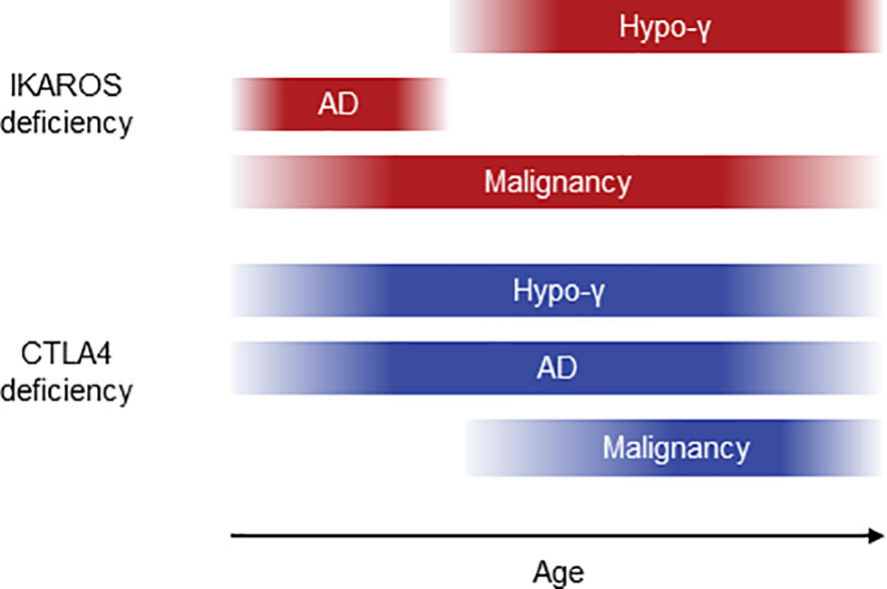
Schematic diagram of clinical courses in IKAROS and CTLA4 deficiencies. AD, autoimmune disease; hypo-γ, hypogammaglobulinemia.

We performed systematic literature reviews of IKAROS and CTLA4 deficiencies and revealed a possibility that the onset of AD was earlier than that of hypogammaglobulinemia in IKAROS deficiency. Furthermore, we demonstrated the more frequent remission of AD before the onset of hypogammaglobulinemia in IKAROS deficiency in this the retrospective longitudinal study. However, such observations were not found in CTLA4 deficiency (**Figure 5**). Several longitudinal studies of IEI have been described, evaluating clinical presentations, quality of life, and psychological status (24, 25). However, to the best of our knowledge, this is the first longitudinal study to focus on the clinical course of complications in IKAROS and CTLA4 deficiencies over time.

The cumulative incidences of hypogammaglobulinemia and AD are 67%-86% and 3%-33% in IKAROS deficiency and 20%-71% and 61%-86% in CTLA4 deficiency, respectively (3, 4, 7, 8, 23). Most of the previous studies showed a cumulative incidence. However, attention should be paid to the incidence when applying it in clinical settings. It does not reflect disease activity or remission, and patients who remain asymptomatic because of their young age are not considered. Furthermore, that incidence cannot be compared between cohorts of patients of different ages. Previous studies showed various cumulative incidences even in the same disease. However, longitudinal evaluation could provide more useful information in the clinical setting. Our study indicated that the onset of AD was more frequent before that of hypogammaglobulinemia in IKAROS deficiency and after hypogammaglobulinemia onset in CTLA4 deficiency. This observation agrees with the result of the systematic literature review, showing no increase in the cumulative incidences of AD at older ages in IKAROS deficiency. This also agrees with the higher number of AD per patient in CTLA4 deficiency. Furthermore, AD remission was more frequent before the onset of hypogammaglobulinemia in IKAROS deficiency but not in CTLA4 deficiency. These observations support the fact that patients with IKAROS deficiency develop AD and hypogammaglobulinemia in that order and achieve AD remission before hypogammaglobulinemia onset. However, patients with CTLA4 deficiency are more likely to develop AD with increasing age than with developing hypogammaglobulinemia.

Our study clarified the problems of literature reviews and cross-sectional studies, and highlighted that change in clinical manifestations over time had not been noticed until now, despite useful information in clinical settings. Our longitudinal study of the small cohort revealed a new feature that large cross-sectional studies would not reveal. Longitudinal studies are useful in evaluating the clinical picture of diseases with a few patients, such as IEI. Notably, these results do not deny the significance of literature reviews and cross-sectional studies. Each has its strengths and limitations, and a further understanding of the diseases can be obtained by their combination (**Supplemental Table 1**).

Our study also provides an important suggestion for AD pathogenesis. AD pathogenesis in IKAROS deficiency remains unclear. Studies in mice have shown that impaired Ikaros function can cause B-cell hyperactivation, upregulation of inflammatory gene programs in T cells, and impaired Treg function, potentially leading to AD (26–28). The evidence shown in this study suggest a prominent role of B cells in AD associated with IKAROS deficiency. The high incidence of systemic AD and detectable autoantibodies and amelioration of AD after hypogammaglobulinemia onset was shown. This hypothesis is also supported by a case report with IKAROS deficiency-associated ITP and autoimmune hepatitis successfully treated by depleting B cells with rituximab (29). Furthermore, B-cell hyperactivation has been described in humans with IKAROS deficiency and mouse models (30). Mice with specific *Ikzf1* deletion in mature B cells, which develops an autoimmune phenotype, show B-cell hyperactivation due to impaired regulation of B-cell receptor anergy and Toll-like receptor signaling (27). Early mouse studies mainly focused on the role of Ikaros in early B-cell development. In humans with IKAROS deficiency, hypogammaglobulinemia also results from impaired early B-cell development (3, 4). However, IKAROS is also involved in B-cell development and function at various stages, suggesting that dysfunction of mature B cells due to IKAROS deficiency could be involved in AD (31). Given this hypothesis, after the progression of hypogammaglobulinemia, early B-cell development arrest causes loss of autoreactive B cells and normal mature B cells, leading to the amelioration of AD. However, we found one patient (patient 8.1) with IKAROS deficiency in our cohort developed rheumatoid arthritis 33 years after hypogammaglobulinemia onset, suggesting additional mechanisms, such as impaired T-cell function as described in mouse models, in addition to impaired B-cell function (26–28). Regarding CTLA4 deficiency, we found a more organ-specific AD and no association between AD remission and hypogammaglobulinemia onset. These observations support a major role of T cells in AD pathogenesis due to impaired Treg function and hyperactivation of effector T cells. Furthermore, our observation of the amelioration of hypogammaglobulinemia after abatacept treatment in patient 15.1 suggests a direct role of activated T cells in the hypogammaglobulinemia associated with CTLA4 deficiency. Although detailed immunological examination, including bone marrow examination, was not performed in this patient, bone marrow infiltration of T cells, which could lead to hypogammaglobulinemia, has been described in previous studies (7, 9).

Given the observations in this study and previous reports, for AD associated with IKAROS deficiency, even refractory AD such as SLE, there is a possibility that treatment is unnecessary after hypogammaglobulinemia onset. B-cell depletion therapy, such as rituximab, may be a good therapeutic option. As described in SLE, belimumab, daratumumab, or CD19-targeted chimeric antigen receptor T-cell therapy might also be useful (32, 33). For AD associated with CTLA4 deficiency, it is necessary to decide the timing of abatacept or HSCT in considering possible treatment-refractory or potential additional AD, as described in a retrospective world survey (9).

Although the reason is unknown, various clinical manifestations can be observed in patients with IKAROS deficiency, including hypogammaglobulinemia, AD, malignancy, and pancytopenia (3, 4). The genotype-phenotype relationship is not established, except for DN N159S and N159T variants, which cause early-onset combined immunodeficiency (5). AD has not been reported in patients with IKAROS DN, which is likely due to severe B-cell deficiency early after birth. We have previously reported patient 6.1 with the Y210C variant, who had transient pancytopenia early after birth (4). We followed up with this patient up to 6 years of age without additional complications in this study. We also found one patient with the R143W variant (patient 10.1). This variant has been reported in two unrelated families, but its effect is controversial (34, 35). One variant was observed in four patients who presented with hypogammaglobulinemia, AD, or both, described as HI (34). Another was in a patient with hypogammaglobulinemia, autoimmune hemolytic anemia, and pancytopenia, described as partial DN (35). However, our patient presented with SLE and APS, typical manifestations as HI. We also found one patient with the R143W variant who presented with hypogammaglobulinemia in our other French cohort. Therefore, from our clinical observations, the R143W variant seems to behave as an HI variant rather than partial DN. The future challenge for IKAROS deficiency is to uncover the clinical course over time and its mechanisms in each variant.

Conclusively, this study provided more realistic manifestations of IKAROS and CTLA4 deficiencies that suggest their pathogenesis. The establishment and use of disease registries and databases will lead to the higher quality of clinical studies and further understanding of human diseases.

## Data Availability Statement

The original contributions presented in the study are included in the article/**Supplementary Material**. Further inquiries can be directed to the corresponding author.

## Ethics Statement

The studies involving human participants were reviewed and approved by the Tokyo Medical and Dental University. Written informed consent to participate in this study was provided by the participants’ legal guardian/next of kin.

## Author Contributions

AH did conception and design. ET, KO, MiY, KK, GY, DK, YY-S, JK, YO, TaI, MN, MaY, KT, ToI, KM-S, YS, TD, TY, YN provided clinical information. AH performed experiment. AH analyzed data and wrote the manuscript. NM, MoY, MT, KI, SN, TM provided critical discussion. SL and HK supervised the study and edited the manuscript. All authors contributed to the article and approved the submitted version.

## Funding

SL is a senior scientist at the Centre National de la Recherche Scientifique (France). The laboratory of SL receives fundings and supports from the Ligue Contre le Cancer-Equipe Labelisée (France), Institut National de la Santé et de la Recherche Médicale (France), the French Foundation of Rare Diseases (France), the Agence Nationale de Recherche ANR (France) (ANR-14-CE14-0028-01, ANR-18-CE15-0025-01 to SL and ANR-10-IAHU-01 to Institut Imagine), the Institut National du Cancer (France) (INCa-PEDIAC-2020, PLBIO20-121), the Société Française de Lutte contre les Cancers et Leucémies de l’Enfant et de l’Adolescent, AREMIG (France), and the Fédération Enfants et Santé (France).

## Conflict of Interest

The authors declare that the research was conducted in the absence of any commercial or financial relationships that could be construed as a potential conflict of interest.

## Publisher’s Note

All claims expressed in this article are solely those of the authors and do not necessarily represent those of their affiliated organizations, or those of the publisher, the editors and the reviewers. Any product that may be evaluated in this article, or claim that may be made by its manufacturer, is not guaranteed or endorsed by the publisher.
