# Supplementary material for: Clinical Courses of IKAROS and CTLA4 Deficiencies: A Systematic Literature Review and Retrospective Longitudinal Study
Source: Front Immunol. 2022 Jan 11;12:784901. doi: 10.3389/fimmu.2021.784901 (PMC8787285; doi:10.3389/fimmu.2021.784901)
Supplement: Supplementary file 1 [file DataSheet_1.docx]

Supplementary material for " **Clinical courses of IKAROS and CTLA4 deficiencies: A systematic literature review and retrospective longitudinal study** " in Frontiers in Immunology

Akihiro Hoshino, Etsushi Toyofuku, Noriko Mitsuiki, Motoi Yamashita, Keisuke Okamoto, Michio Yamashita, Kenji Kanda, Genki Yamato, Dai Keino, Yuri Yoshimoto-Suzuki, Junji Kamizono, Yasuhiro Onoe, Takuya Ichimura, Mika Nagao, Masaru Yoshimura, Koji Tsugawa, Toru Igarashi, Kanako Mitsui-Sekinaka, Yujin Sekinaka, Takehiko Doi, Takahiro Yasumi, Yozo Nakazawa, Masatoshi Takagi, Kohsuke Imai, Shigeaki Nonoyama, Tomohiro Morio, Sylvain Latour, and Hirokazu Kanegane

Corresponding author

Hirokazu Kanegane MD, PhD

Department of Child Health and Development, Graduate School of Medical and Dental Sciences, Tokyo Medical and Dental University, Tokyo, Japan

E-mail: hkanegane.ped@tmd.ac.jp

**Supplementary Table 1. Comparisons of study methods.**

|  | Strengths | Limitations |
| --- | --- | --- |
| Longitudinal study | - Evaluation of changes within individuals over time  - Advantages for diseases with a small number of individuals in retrospective studies | - Inefficiency of time and cost in prospective studies |
| Cross-sectional study | - Efficiency of time and cost  - Advantages for diseases with a large number of individuals | - No evaluation of changes within individuals over time |

**Supplementary Table 2. Reported patients with IKAROS and CTLA4 deficiencies.**

| Ref. | Numbers of included patients | Numbers of excluded patients |  |
| --- | --- | --- | --- |
| **IKAROS deficiency** | | | |
| 3 | 29 (HI) |  |  |
| 4 | 11 (HI), 1 (DN) |  |  |
| E1 | 1 (HI) |  |  |
| E2 | 1 (DN) |  |  |
| E3 | 1 (HI) |  |  |
| E4 | 3 (HI) |  |  |
| 30 | 2 (HI) |  |  |
| E5 | 1 (HI) | 48 (no details) |  |
| 5 | 7 (DN) |  | 1 overlapped in 4, 1 overlapped in E2 |
| 34 | 6 (HI), 3 (DD) | 2 (not tested), 6 (not determined) |  |
| E6 | 3 (HI) |  |  |
| E7 | 3 (HI) |  |  |
| E8 | 4 (DN) |  | 1 overlapped in E2 and 5, 2 overlapped in 5 |
| E9 | 1 (HI) | 2 (no details) |  |
| 29 | 2 (HI) |  |  |
| E10 | 1 (HI) |  |  |
| E11 | 1 (HI) |  |  |
| 35 | 1 (HI/partial DN) |  |  |
| 6 | 13 (DD) |  |  |
| E12 |  | 20 (no details) | 18 overlapped in 3, 2 overlapped in E4 |
| E13 |  | 1 (no details) |  |
| E14 |  | 16 (not details) | 16 overlapped in 3 |
| E15 |  | 1 (no details) |  |
| 13 |  | 1 (no details) | 1 overlapped in 4 |
| E16 |  | 2 (no details) |  |
| E17 |  | 21 (no details) | 1 overlapped in 3 |
| 14 |  | 4 ( no details) |  |
| E18 |  | 6 (no details) |  |
| 2 |  | 5 (no details) |  |
| E19 |  | 1 (not determined) |  |
| **CTLA4 deficiency** | | | |
| 11 | 133 | 2 (large deletion) |  |
| 8 | 23 |  | 23 overlapped in 11 |
| 7 | 9 |  |  |
| E20 | 3 |  | 3 overlapped in 11 |
| E21 | 9 |  | 6 overlapped in 11 |
| 17 | 1 |  | 1 overlapped in 11 |
| E22 | 1 |  |  |
| E23 | 1 |  |  |
| E24 | 1 |  | 1 overlapped in 8 and 11 |
| E25 | 1 |  | 1 overlapped in 11 |
| E26 | 1 |  | 1 overlapped in 11 |
| E27 | 1 |  |  |
| 23 | 15 |  | 15 overlapped in 11 |
| 16 | 17 |  | 13 overlapped in 11 |
| E28 | 4 |  | 1 overlapped in 16 |
| E29 | 1 |  |  |
| E30 | 1 |  |  |
| E31 | 1 |  |  |
| E32 | 1 |  |  |
| E33 | 3 |  |  |
| E34 | 1 |  |  |
| E35 | 1 |  |  |
| E36 | 4 |  |  |
| E37 | 3 |  | 1 overlapped in E34 |
| 19 | 1 |  | 1 overlapped in 11 |
| E38 | 3 |  |  |
| E39 | 3 |  |  |
| E40 | 3 |  |  |
| E13 |  | 2 (no details) |  |
| E15 |  | 2 (no details) |  |
| E41 |  | 2 (no details) |  |
| E42 |  | 1 (large deletion) |  |
| 18 |  | 15 (no details) | 15 overlapped in 11 |
| E43 |  | 3 (no details), 2 (large deletion) |  |
| 14 |  | 2 (no details) | 2 overlapped in 11 |
| E44 |  | 3 (no details) |  |
| E16 |  | 8 (no details) |  |
| E45 |  | 4 (no details) |  |
| E46 |  | 1 (no details) |  |
| E47 |  | 1 (large deletion) |  |
| 2 |  | 4 (no details) |  |
| E18 |  | 5 (no details) |  |
| 9 |  | 173 (no details), 3 (large deletion) | 135 overlapped in 11, 2 overlapped in E41 |
| E48 |  | 2 (no details) |  |
| E49 |  | 6 (no details) | 3 overlapped in 11 |

**Supplementary Table 3.** **Baseline description of individuals with IKAROS deficiency.**

|  | IKAROS HI | IKAROS DD | IKAROS DN |
| --- | --- | --- | --- |
| Number of patients | 66 | 16 | 8 |
| Sex (M/F) | 34/32 (n =66) | 8/8 (n = 16) | 6/1 (n = 7) |
| Age at last follow-up | 23 [12-43] (n = 61) | 38 [21-50] (n = 15) | 10 [3-19] (n = 8) |
| Age at onset | 10 [4-19] (n = 49) | 9 [7-21] (n = 6) | 0.6 [0.3-1] (n = 8) |
| Age at onset of hypo-γ | 10 [6-19] (n = 36) | 10 [9-28] (n = 3) | 0.6 [0.3-1] (n = 8) |
| Age at onset of AD | 7 [3-14] (n = 16) | 11 [9-31] (n = 4) | (n = 0) |
| Age at onset of malignancy | 4 [3-5] (n = 4) | 6 (n = 2) | 13 (n = 1) |

The median ages are shown [with 25th and 75th percentiles] (year).

AD, autoimmune disease; hypo-γ, hypogammaglobulinemia.

**Supplementary Table 4. Autoimmune diseases described in IKAROS deficiency.**

| Ref. | Patient | Variant | Effect | AD | Age at onset of AD (year) | Autoantibody | IgG/IgA/IgM (mg/dL) |  |
| --- | --- | --- | --- | --- | --- | --- | --- | --- |
| 3 | A2 | p.R162L | HI | ITP | 3 | ND | *434/ND/ND** | **5 yo* |
| 4 | A.1 | p.C147R | HI | ITP | 5 | platelet-associated IgG | 784/2/9 |  |
| 4 | D.I.1 | p.R162Q | HI | ITP | 20 | ND | *1136/204/79** | **68 yo* |
| 4 | D.III.1 | p.R162Q | HI | IgA vasculitis | 3 | ND | 824/87/111 |  |
| 4 | E.II.1 | c.589+1G>A | HI | SLE | 3 | ANA, anti-dsDNA, anti-cardiolipin, anti-ribonucleoprotein | 2329/154/97 |  |
| 29 | P2 | p.L188V | HI | SLE, APS | 12 | ANA, anti-dsDNA, anti-cardiolipin IgG/IgM, LA | 938/60/90 |  |
| E4 | II.3 | p.S46fs | HI | JIA | 6 | negative | *700/ND/ND** | **9 yo* |
| E4 | II.4 | p.S46fs | HI | myasthenia gravis | 12 | anti-acetylcholine receptor | *1270/ND/40** | **11 yo* |
| E6 | Patient | p.H195R | HI | ITP | 4 | negative | 444/41/59 |  |
| E6 | Mother | p.H196R | HI | ITP | 21 | negative | *997/54/21** | **44 yo* |
| 33 | A.I.2 | p.R143W | HI/partial DN | Hashimoto thyroiditis | ND | ND | *113/28/28** | **53 yo* |
| 33 | B.II.1 | p.M494fs | DD | ANCA-negative vasculitis | 37 | ND | 438/54/24 |  |
| E10 |  | p.H163fs | HI | IgA vasculitis | 8 | ANA, deposition of IgA in the dermal vessels on DIF | 1210/242/126 |  |
| E9 |  | p.D120V | HI | SLE | 14 | anti-dsDNA, anti-Sm | norm/norm/norm |  |
| 28 |  | p.R162W | HI | ITP | 1.5 |  | *681/129/<10** | **7 yo* |
|  |  |  |  | autoimmune hepatitis | 6 | ANA, anti-LKM-1 | *681/129/<10** | **7 yo* |
| E7 | I.1 | p.R167R | HI | ITP | childhood | *ANA** | *790/ND/ND** | **57 yo* |
| E7 | II.1 |  | HI | APS | childhood | ANA, anti-cardiolipin IgG/M, LA | ND/ND/ND |  |
| E7 | II.2 |  | HI | SLE, APS | 11 | ANA, anti-dsDNA, anti-cardiolipin IgG/M, LA | *610/114/49** | **24 yo* |
| 34 |  | p.R143W | HI/partial DN | AIHA | 0.25 | direct Coobms | 395/ND/31.9 |  |
| 6 | A.III.1 | p.R213* | DD | ITP | 9 | ND | *316/23/45** | **10 yo* |
| 6 | C.III.1 | p.C467R | DD | ITP | 9 | ND | 611/37/32 |  |
|  |  |  |  | AIHA | 13 | ND | *412/16/24** | **During the post-rituximab follow-up* |
| 6 | C.III.2 | p.C467R | DD | Hashimoto thyroiditis | ND | ND | ND/ND/ND |  |
| 6 | D.II.1 | p.R502L | DD | ITP, neutropenia | 12 | ND | 730/115/100 |  |

*Italic numbers indicate serum immunoglobulin levels measured not at the onset of autoimmune disease.

AD, autoimmune disease; AIHA, autoimmune hemolytic anemia; ANA, antinuclear antibody; ANCA, antineutrophil cytoplasm antibodies; APS, antiphospholipid syndrome; DIF, direct immunofluorescence; JIA, juvenile idiopathic arthritis; ITP, immune thrombocytopenia; LA, lupus anticoagulant; LKM-1, liver kidney microsome type 1; ND, not determined; norm, normal; SLE, systemic lupus erythematosus.

**Supplementary Table 5. Patients with IKAROS and CTLA4 deficiencies in our cohort.**

| Patient | Variant | Effect | Sex | Follow-up period | Clinical manifestation | Treatment | Ref. |
| --- | --- | --- | --- | --- | --- | --- | --- |
| **IKAROS deficiency** | | | | | | | |
| 1.1 | p.C147R | HI | M | 13 y 7 m | hypo-γ, ITP | IVIG | 4 (A.1) |
| 2.1 | p.N159S | DN | F | 19 y (died of pneumonia) | hypo-γ, CID | IVIG | 4 (B.1), 5 |
| 3.1 | p.R162W | HI | M | 43 y 6 m | hypo-γ | IVIG | 4 (C.I.1) |
| 3.2 | p.R162W | HI | F | 17 y 11m | hypo-γ | IVIG | 4 (C.II.1) |
| 3.3 | p.R162W | HI | M | 16 y 3 m | hypo-γ | IVIG | 4 (C.II.2) |
| 4.1 | p.R162Q | HI | F | 74 y 0 m | ITP | corticosteroid | 4 (D.I.1) |
| 4.2 | p.R162Q | HI | F | 48 y 8 m | AS | none | 4 (D.II.1) |
| 4.3 | p.R162Q | HI | M | 17 y 8 m | hypo-γ, IgA vasculitis | IVIG | 4 (D.III.1) |
| 5.1 | c.589+1G>A | HI | F | 38 y | AS | none | 4 (E.I.1) |
| 5.2 | c.589+1G>A | HI | M | 14 y 4 m | hypo-γ, SLE | IVIG, corticosteroid, MMF, immunoadsorption | 4 (E.II.1), 14 |
| 6.1 | p.Y210C | HI | M | 8 y 5 m | AS | none | 4 (F.1) |
| 6.2 | p.Y210C | HI | M | 6 y 0 m | transient pancytopenia | blood transfusion | 4 (F.2) |
| 7.1 | p.K157del | HI | F | 15 y 8 m | hypo-γ | IVIG | 15 |
| 8.1 | p.F490del | DD | F | 47 y 8 m (died of sepsis) | hypo-γ, RA | IVIG, corticosteroid, TAC, abatacept | 15 |
| 9.1 | p.H508Y | DD | F | 27 y 1 m | hypo-γ, cutaneous leukocytoclastic vasculitis | IVIG | 15 |
| 10.1 | p.R143W | HI | M | 18 y 11 m | SLE, APS | corticosteroid, MMF | 15 |
| **CTLA4 deficiency** | | | | | | | |
| 11.1 | p.F28fs |  | M | 36 y 3 m | hypo-γ, enteropathy, T1DM, CNS involvement, gastric adenocarcinoma | IVIG, insulin, gastrectomy | 9, 11 (M.II.3), 16, 17 |
| 12.1 | p.Y177* |  | F | 76 y 9 m | AS | none | 9, 11 (N.I.2) |
| 12.2 | p.Y177* |  | F | 52 y 6 m | AS | none | 9, 11 (N.II.1) |
| 12.3 | p.Y177* |  | M | 47 y 11m | AS | none | 9, 11 (N.II.3) |
| 12.4 | p.Y177* |  | M | 17 y 3 m (HSCT) | hypo-γ, ITP, IgA vasculitis, AIN, CNS involvement, GLILD | IVIG, corticosteroid, HSCT | 9, 11 (N.III.2) |
| 13.1 | p.W165* |  | M | 38 y 7 m | alopecia | none | 9, 11 (U.I.1), 18 |
| 13.2 | p.W165* |  | M | 15 y 0 m | hypo-γ, ITP, CNS involvement, GLILD | IVIG, corticosteroid, TAC, sirolimus, rituximab, abatacept | 9, 11 (U.II.1), 14, 18 |
| 13.3 | p.W165* |  | M | 11 y 10 m | alopecia | none | 9, 11 (U.II.2), 18 |
| 13.4 | p.W165* |  | F | 10 y 8 m | T1DM | insulin | 9, 11 (U.II.3), 18 |
| 14.1 | p.G146R |  | F | 19 y 5 m | AIHA, ITP, AIN, IgA vasculitis | corticosteroid | 9, 11 (V.II.1), 14 |
| 15.1 | p.Q12* |  | M | 48 y 8 m | hypo-γ, ITP, AIHA, granulomatous interstitial nephritis, enteropathy | corticosteroid, abatacept | 9, 11 (W.I.1), 18 |
| 15.2 | p.Q12* |  | M | 22 y 1 m | hypo-γ, ITP | IVIG, corticosteroid | 9, 11 (W.II.1) |
| 15.3 | p.Q12* |  | F | 14 y 1 m (HSCT) | hypo-γ, GLILD, alopecia, AIHA, enteropathy | IVIG, corticosteroid, CyA, TAC, HSCT | 9, 11 (W.II.2), 18 |
| 15.4 | p.Q12* |  | F | 8 y 7 m (HSCT) | AIHA, alopecia, autoimmune hepatitis, ITP | IVIG, corticosteroid, TAC, HSCT | 9, 11 (W.II.3), 18 |
| 16.1 | p.G52V |  | M | 60 y 10 m | AS | none | 9, 11 (AA.III.3), 18 |
| 16.2 | p.G52V |  | M | 21 y 2 m (HSCT) | hypo-γ, alopecia, GLILD, CNS involvement | IVIG, corticosteroid, TAC, abatacept, HSCT | 9, 11 (AA.IV.1), 18 |
| 17.1 | p.V40A |  | F | 51 y | hypothyroidism | levothyroxine | 9, 11 (AB.I.2), 18 |
| 17.2 | p.V40A |  | F | 26 y | hyperthyroidism | antithyroid drug | 9, 11 (AB.II.1) |
| 17.3 | p.V40A |  | F | 22 y 1 m (died of liver failure) | enteropathy, ITP, AIHA | corticosteroid, 6-mercaptopurine | 9, 11 (AB.II.2), 18, 19 |
| 18.1 | p.N14fs |  | F | 49 y 3 m | hypo-γ, alopecia | IVIG, corticosteroid | 9, 11 (AC.II.1), 18 |
| 19.1 | p.D78fs |  | F | 44 y | AS | none | 9, 11 (AD.I.2), 18 |
| 19.2 | p.D78fs |  | M | 22 y 3 m | ITP, AIHA | IVIG, corticosteroid, plasmapheresis | 9, 11 (AD.II.1), 18 |
| 20.1 | p.V40M |  | F | 21 y 1 m | hypo-γ, AIN, AIHA | IVIG, corticosteroid | 18 |
| 21.1 | p.C85Y |  | M | 64 y | AS | none |  |
| 21.2 | p.C85Y |  | F | 28 y 6 m | hypo-γ, aplastic anemia | CyA |  |
| 21.3 | p.C85Y |  | M | 28 y 6 m | hypo-γ, enteropathy, interstitial cystitis, drug-induced lupus | IVIG, corticosteroid, sirolimus, infliximab, abatacept |  |
| 22.1 | p.V84A |  | F | 33 y 9 m | hypo-γ, DLBCL | IVIG, chemotherapy | 20 |
| 23.1 | p.F162fs |  | F | 43 y 4 m | hypo-γ, enteropathy, ITP | IVIG |  |
| 24.1 | p.A54T |  | F | 46 y 6 m | hypo-γ, enteropathy, GLILD | IVIG |  |
| 25.1 | p.C129R |  | M | 6 y 8 m | enteropathy, ITP, AIN | corticosteroid |  |
| 26.1 | p.R51* |  | F | 47 y 2 m | hypo-γ, GLILD, enteropathy | IVIG, 5-aminosalicylic acid |  |

AIHA, autoimmune hemolytic anemia; AIN, autoimmune neutropenia; APS, antiphospholipid syndrome; AS, asymptomatic; CID, combined immunodeficiency; CNS, central nervous system; CyA, cyclosporine A; DLBCL, diffuse large B cell lymphoma; GLILD, granulomatous lymphocytic interstitial lung disease; hypo-γ, hypogammaglobulinemia; ITP, immune thrombocytopenia; IVIG, intravenous immunoglobulin; MMF, mycophenolate mofetil; RA, rheumatoid arthritis; SLE, systemic lupus erythematosus; TAC, tacrolimus; T1DM, type 1 diabetes mellitus.


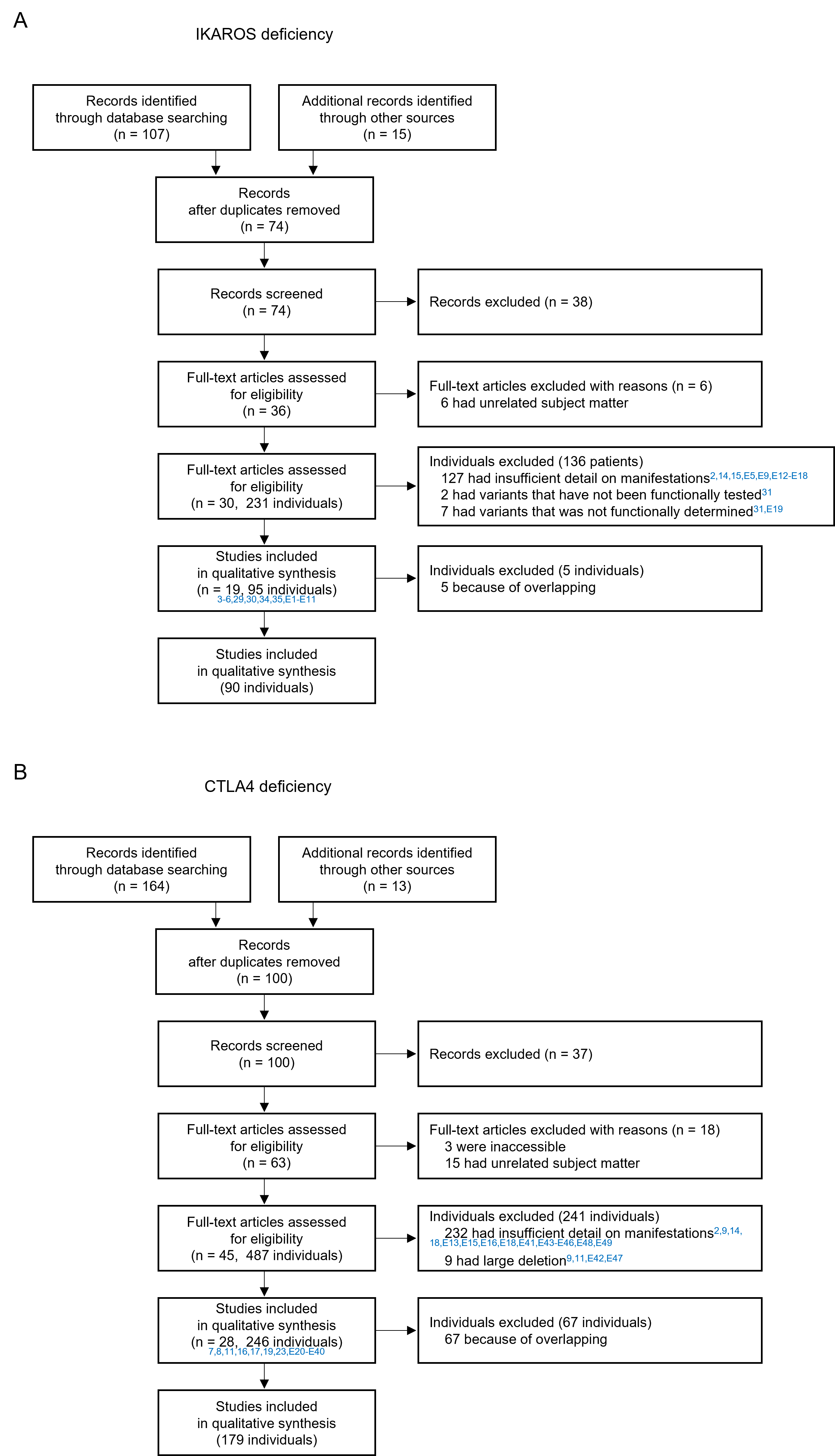


**Supplementary Figure 1. Flow Chart showing the article identification, screening, eligibility and inclusion process.**


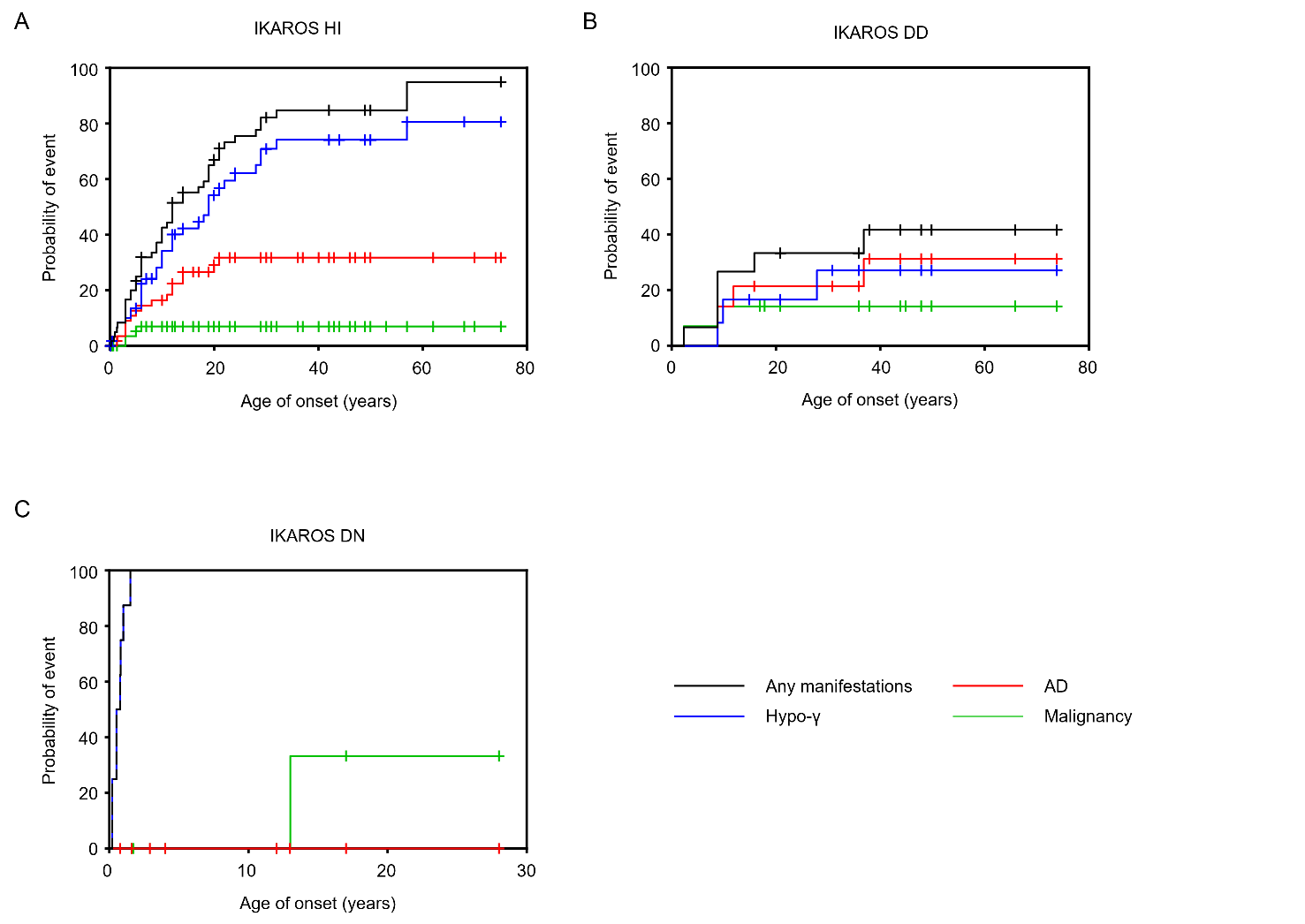


**Supplemental Figure 2.** Kaplan-Meier curves. **(A)** Cumulative incidence of any manifestations (n = 62), hypogammaglobulinemia (n = 61), autoimmune disease (n = 58) and malignancy (n = 61) in IKAROS HI. **(B)** Cumulative incidence of any manifestations (n = 15), hypogammaglobulinemia (n = 12), autoimmune disease (n = 14) and malignancy (n = 14) in IKAROS DD. **(C)** Cumulative incidence of any manifestations (n = 8), hypogammaglobulinemia (n = 8), autoimmune disease (n = 8) and malignancy (n = 8) in IKAROS DN. AD, autoimmune disease; hypo-γ, hypogammaglobulinemia.


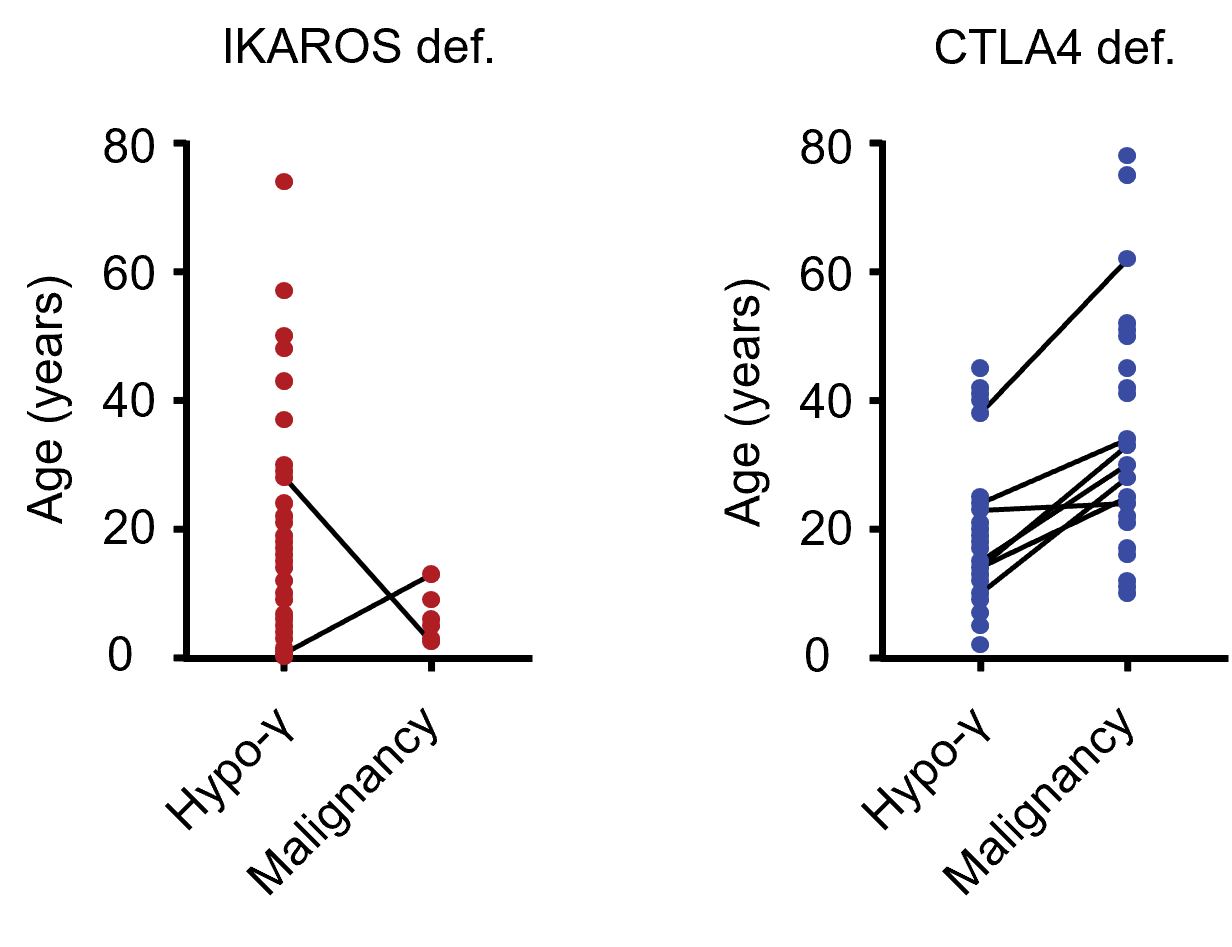


**Supplemental Figure 3. Comparison of age of the onset.**

Hypo-γ, hypogammaglobulinemia.


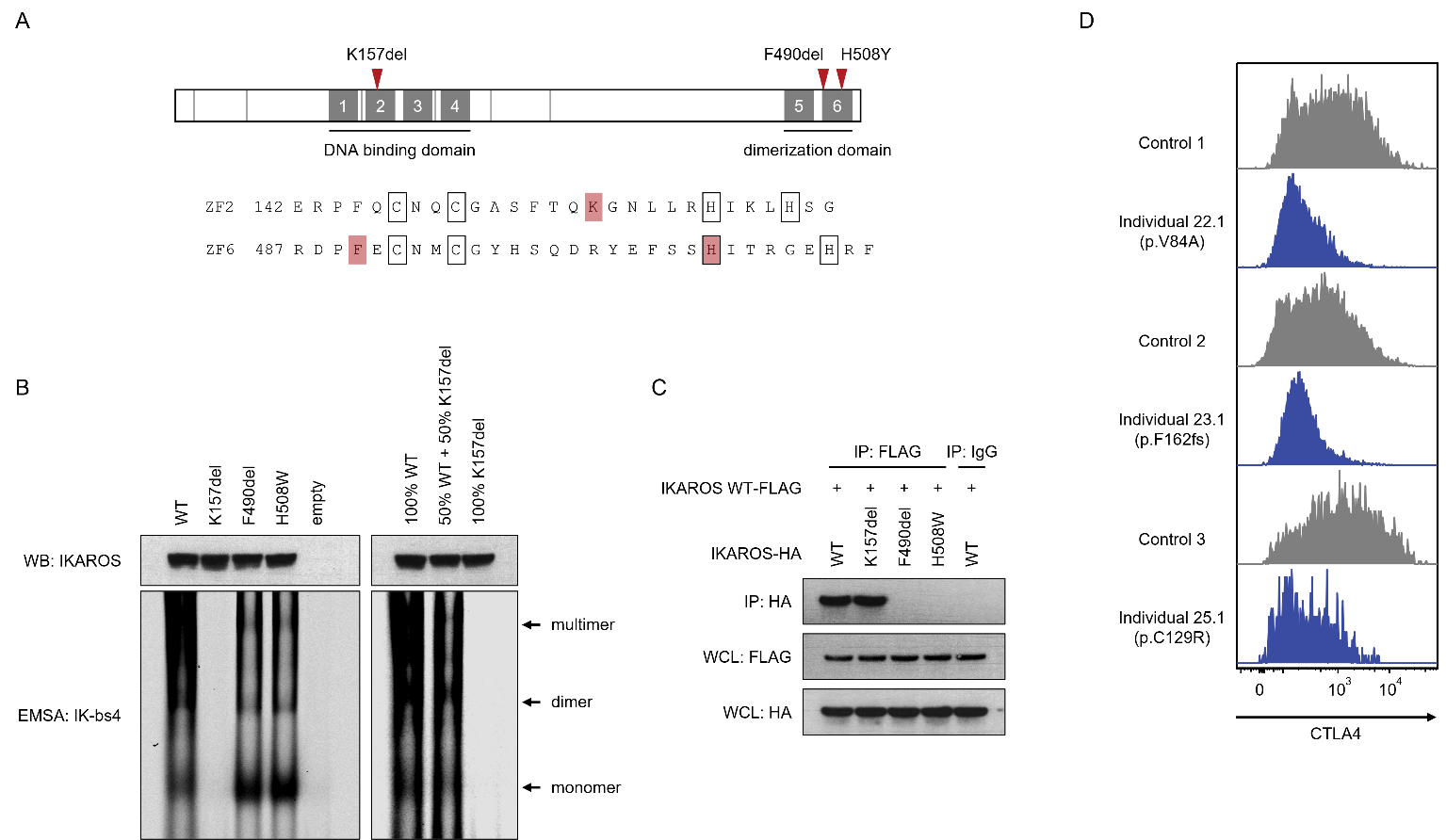


**Supplemental Figure 4. Loss of function variants revealed by functional assay. (A)** IKAROS protein structure and sequence of zinc fingers 2 and 6. Letters in squares indicate Cys (C) and His (H) residues, which coordinate the zinc atom. **(B)** Electrophoresis mobility shift assay and immunoblot assessing DNA-binding ability in IKAROS mutants. **(C)** Immunoprecipitation assessing dimerization ability in IKAROS mutants. **(D)** Flow cytometry assessing CTLA4 protein expression in CD4^+^FOXP3^+^ T cells.

**Supplemental references**

E1. Goldman FD, Gurel Z, Al-Zubeidi D, Fried AJ, Icardi M, Song C, Dovat S. Congenital pancytopenia and absence of B lymphocytes in a neonate with a mutation in the Ikaros gene. *Pediatr Blood Cancer* (2012) 58:591–597. doi:10.1002/pbc.23160

E2. Yoshida N, Sakaguchi H, Muramatsu H, Okuno Y, Song C, Dovat S, Shimada A, Ozeki M, Ohnishi H, Teramoto T, et al. Germline IKAROS mutation associated with primary immunodeficiency that progressed to T-cell acute lymphoblastic leukemia. *Leukemia* (2017) 31:1221–1223. doi:10.1038/leu.2017.25

E3. Chen Q, Wang X, Wang W, Zhou Q, Liu D, Wang Y. B-cell Deficiency: A de novo IKZF1 patient and review of the literature. *J Investig Allergol Clin Immunol* (2018) 28:53–56. doi:10.18176/jiaci.0207

E4. Bogaert DJ, Kuehn HS, Bonroy C, Calvo KR, Dehoorne J, Vanlander AV, De Bruyne M, Cytlak U, Bigley V, De Baets F, et al. A novel IKAROS haploinsufficiency kindred with unexpectedly late and variable B-cell maturation defects. *J Allergy Clin Immunol* (2018) 141:432–435.e7. doi:10.1016/j.jaci.2017.08.019

E5. Churchman ML, Qian M, te Kronnie G, Zhang R, Yang W, Zhang H, Lana T, Tedrick P, Baskin R, Verbist K, et al. Germline genetic IKZF1 variation and predisposition to childhood acute lymphoblastic leukemia. *Cancer Cell* (2018) 33:937–948.e8. doi:10.1016/j.ccell.2018.03.021

E6. Sriaroon P, Chang Y, Ujhazi B, Csomos K, Joshi HR, Zhou Q, Close DW, Walter JE, Kumánovics A. Familial immune thrombocytopenia associated with a novel variant in IKZF1. *Front Pediatr* (2019) 7:139. doi:10.3389/fped.2019.00139

E7. Dieudonné Y, Guffroy A, Vollmer O, Carapito R, Korganow A-S. IKZF1 Loss-of-function variant causes autoimmunity and severe familial antiphospholipid syndrome. *J Clin Immunol* (2019) 39:353–357. doi:10.1007/s10875-019-00643-2

E8. Kellner ES, Krupski C, Kuehn HS, Rosenzweig SD, Yoshida N, Kojima S, Boutboul D, Latour S, Barlogis V, Galambrun C, et al. Allogeneic hematopoietic stem cell transplant outcomes for patients with dominant negative IKZF1/IKAROS mutations. *J Allergy Clin Immunol* (2019) 144:339–342. doi:10.1016/j.jaci.2019.03.025

E9. Belot A, Rice GI, Omarjee SO, Rouchon Q, Smith EMD, Moreews M, Tusseau M, Frachette C, Bournhonesque R, Thielens N, et al. Contribution of rare and predicted pathogenic gene variants to childhood-onset lupus: a large, genetic panel analysis of British and French cohorts. The *Lancet Rheumatol* (2020) 2:e99–e109. doi:10.1016/S2665-9913(19)30142-0

E10. Banday AZ, Jindal AK, Kaur A, Saka R, Parwaiz A, Sachdeva MUS, Rawat A. Cutaneous IgA vasculitis—presenting manifestation of a novel mutation in the IKZF1 gene. *Rheumatology* (2021) 60:e101–e103. doi:10.1093/rheumatology/keaa492

E11. Kuehn HS, Gloude NJ, Dimmock D, Tokita M, Wright M, Rosenzweig SD, Collins C. Abnormal SCID newborn screening and spontaneous recovery associated with a novel haploinsufficiency IKZF1 mutation. *J Clin Immunol* (2021) 41:1241–1249. doi:10.1007/s10875-021-01035-1

E12. Cytlak U, Resteu A, Bogaert D, Kuehn HS, Altmann T, Gennery A, Jackson G, Kumanovics A, Voelkerding KV, Prader S, et al. Ikaros family zinc finger 1 regulates dendritic cell development and function in humans. *Nat Commun* (2018) 9:1239. doi:10.1038/s41467-018-02977-8

E13. Maffucci P, Filion CA, Boisson B, Itan Y, Shang L, Casanova J-L, Cunningham-Rundles C. Genetic Diagnosis Using Whole exome sequencing in common variable immunodeficiency. *Front Immunol* (2016) 7: doi:10.3389/fimmu.2016.00220

E14. Abdulhay N, Fiorini C, Kumánovics A, Sun AA, Hansen-Rejali J, Voelkerding KV, Rosenzweig SD, Hill HR, Sankaran VG. Normal hematologic parameters and fetal hemoglobin silencing with heterozygous IKZF1 mutations. *Blood* (2016) 128:2100–2103. doi:10.1182/blood-2016-08-731943

E15. Stray-Pedersen A, Sorte HS, Samarakoon P, Gambin T, Chinn IK, Coban Akdemir ZH, Erichsen HC, Forbes LR, Gu S, Yuan B, et al. Primary immunodeficiency diseases: Genomic approaches delineate heterogeneous Mendelian disorders. *J Allergy Clin Immunol* (2017) 139:232–245. doi:10.1016/j.jaci.2016.05.042

E16. Hadjadj J, Aladjidi N, Fernandes H, Leverger G, Magérus-Chatinet A, Mazerolles F, Stolzenberg M-C, Jacques S, Picard C, Rosain J, et al. Pediatric Evans syndrome is associated with a high frequency of potentially damaging variants in immune genes. *Blood* (2019) 134:9–21. doi:10.1182/blood-2018-11-887141

E17. Pastorczak A, Hogendorf A, Urbanska Z, Budzynska E, Jesionek‐Kupnicka D, Gach A, Hawula W, Smigiel R, Skiba P, Sasiadek M, et al. Broad phenotypic spectrum of germ line 7p12.1 microdeletions encompassing the IKZF1 gene includes predisposition to acute lymphoblastic leukemia. *Genes Chromosomes Cancer* (2021) 60:79–87. doi:10.1002/gcc.22914

E18. Thaventhiran JED, Lango Allen H, Burren OS, Rae W, Greene D, Staples E, Zhang Z, Farmery JHR, Simeoni I, Rivers E, et al. Whole-genome sequencing of a sporadic primary immunodeficiency cohort. *Nature* (2020) 583:90–95. doi:10.1038/s41586-020-2265-1

E19. Brodie SA, Khincha PP, Giri N, Bouk AJ, Steinberg M, Dai J, Jessop L, Donovan FX, Chandrasekharappa SC, de Andrade KC, et al. Pathogenic germline IKZF1 variant alters hematopoietic gene expression profiles. *Cold Spring Harb Mol Case Stud* (2021) 7:a006015. doi:10.1101/mcs.a006015

E20. Zeissig S, Petersen B-S, Tomczak M, Melum E, Huc-Claustre E, Dougan SK, Laerdahl JK, Stade B, Forster M, Schreiber S, et al. Early-onset Crohn’s disease and autoimmunity associated with a variant in CTLA-4. *Gut* (2015) 64:1889–1897. doi:10.1136/gutjnl-2014-308541

E21. Slatter MA, Engelhardt KR, Burroughs LM, Arkwright PD, Nademi Z, Skoda-Smith S, Hagin D, Kennedy A, Barge D, Flood T, et al. Hematopoietic stem cell transplantation for CTLA4 deficiency. *J Allergy Clin Immunol* (2016) 138:615–619.e1. doi:10.1016/j.jaci.2016.01.045

E22. Shields CL, Say EAT, Mashayekhi A, Garg SJ, Dunn JP, Shields JA. Assessment of CTLA-4 deficiency–related autoimmune choroidopathy response to abatacept. *JAMA Ophthalmol* (2016) 134:844. doi:10.1001/jamaophthalmol.2016.1013

E23. Lee S, Moon JS, Lee C-R, Kim H-E, Baek S-M, Hwang S, Kang GH, Seo JK, Shin CH, Kang HJ, et al. Abatacept alleviates severe autoimmune symptoms in a patient carrying a de novo variant in CTLA-4. *J Allergy Clin Immunol* (2016) 137:327–330. doi:10.1016/j.jaci.2015.08.036

E24. Greil C, Roether F, La Rosée P, Grimbacher B, Duerschmied D, Warnatz K. Rescue of cytokine storm due to HLH by hemoadsorption in a CTLA4-deficient patient. *J Clin Immunol* (2017) 37:273–276. doi:10.1007/s10875-017-0377-7

E25. Kucuk ZY, Charbonnier L-M, McMasters RL, Chatila T, Bleesing JJ. CTLA-4 haploinsufficiency in a patient with an autoimmune lymphoproliferative disorder. *J Allergy Clin Immunol* (2017) 140:862–864.e4. doi:10.1016/j.jaci.2017.02.032

E26. Navarini AA, Hruz P, Berger CT, Hou TZ, Schwab C, Gabrysch A, Higgins R, Frede N, Padberg Sgier B-C, Kämpe O, et al. Vedolizumab as a successful treatment of CTLA-4–associated autoimmune enterocolitis. *J Allergy Clin Immunol* (2017) 139:1043–1046.e5. doi:10.1016/j.jaci.2016.08.042

E27. Moraes-Fontes MF, Hsu AP, Caramalho I, Martins C, Araújo AC, Lourenço F, Taulaigo AV, Lladó A, Holland SM, Uzel G. Fatal CTLA-4 heterozygosity with autoimmunity and recurrent infections: a de novo mutation. *Clin Case Rep* (2017) 5:2066–2070. doi:10.1002/ccr3.1257

E28. Watson LR, Slade CA, Ojaimi S, Barnes S, Fedele P, Smith P, Marum J, Lunke S, Stark Z, Hunter MF, et al. Pitfalls of immunotherapy: lessons from a patient with CTLA-4 haploinsufficiency. *Allergy Asthma Clin Immunol* (2018) 14:65. doi:10.1186/s13223-018-0272-7

E29. van Leeuwen EM, Cuadrado E, Gerrits AM, Witteveen E, de Bree GJ. Treatment of intracerebral lesions with abatacept in a CTLA4-haploinsufficient patient. *J Clin Immunol* (2018) 38:464–467. doi:10.1007/s10875-018-0511-1

E30. Siggs OM, Russell A, Singh-Grewal D, Wong M, Chan P, Craig ME, O’Loughlin T, Stormon M, Goodnow CC. Preponderance of CTLA4 variation associated with autosomal dominant immune dysregulation in the MYPPPY motif. *Front Immunol* (2019) 10:1544. doi:10.3389/fimmu.2019.01544

E31. Ureshino H, Koarada S, Kamachi K, Yoshimura M, Yokoo M, Kubota Y, Ando T, Ichinohe T, Morio T, Kimura S. Immune dysregulation syndrome with de novo CTLA4 germline mutation responsive to abatacept therapy. *Int J Hematol* (2020) 111:897–902. doi:10.1007/s12185-020-02834-9

E32. Zaremehrjardi F, Baniadam L, Seif F, Arshi S, Bemanian MH, Shokri S, Rezaeifar A, Fallahpour M, Nabavi M. A patient with CTLA-4 Haploinsufficiency with multiple autoimmune presentations: A case report. *Iran J Immunol* (2020) 17:244–249. doi:10.22034/iji.2020.85641.1721

E33. Ayrignac X, Goulabchand R, Jeziorski E, Rullier P, Carra-Dallière C, Lozano C, Portales P, Vincent T, Viallard JF, Menjot de Champfleur N, et al. Two neurologic facets of CTLA4-related haploinsufficiency. *Neurol Neuroimmunol Neuroinflamm* (2020) 7:e751. doi:10.1212/NXI.0000000000000751

E34. Mahat U, Terzioglu MK, Buhtoiarov I. CTLA4 haploinsufficiency as a predisposition to classical Hodgkin lymphoma. *Pediatr Hematol Oncol* (2020) 37:176–183. doi:10.1080/08880018.2019.1710310

E35. Siddiqi AE, Liu AY, Charville GW, Kunder CA, Uzel G, Sadighi Akha AA, Oak J, Martin B, Sacha J, Lewis DB, et al. Disseminated Pneumocystis jirovecii infection with osteomyelitis in a patient with CTLA-4 haploinsufficiency. *J Clin Immunol* (2020) 40:412–414. doi:10.1007/s10875-020-00748-z

E36. Yap JY, Gloss B, Batten M, Hsu P, Berglund L, Cai F, Dai P, Parker A, Qiu M, Miley W, et al. Everolimus-induced remission of classic Kaposi’s sarcoma secondary to cryptic splicing mediated CTLA4 haploinsufficiency. *J Clin Immunol* (2020) 40:774–779. doi:10.1007/s10875-020-00804-8

E37. Mahat U, Ambani NM, Rotz SJ, Radhakrishnan K. Heterozygous CTLA4 splice site mutation c.458-1G > C presenting with immunodeficiency and variable degree of immune dysregulation in three generation kindred of Caribbean descent. *Pediatr Hematol Oncol* (ahead of print) doi:10.1080/08880018.2021.1906802

E38. Yang L, Xue X, Chen X, Wu J, Yang X, Xu L, Tang X, Wang M, Mao H, Zhao X. Abatacept is effective in Chinese patients with LRBA and CTLA4 deficiency. *Genes Dis* (2021) 8:662–668. doi:10.1016/j.gendis.2020.03.001

E39. Lanz A-L, Riester M, Peters P, Schwerd T, Lurz E, Hajji MS, Rohlfs M, Ley-Zaporozhan J, Walz C, Kotlarz D, et al. Abatacept for treatment-refractory pediatric CTLA4-haploinsufficiency. *Clin Immunol* (2021) 229:108779. doi:10.1016/j.clim.2021.108779

E40. Grammatikos A, Johnston S, Rice CM, Gompels M. A family with a novel CTLA4 haploinsufficiency mutation and neurological symptoms. *J Clin Immunol* (2021) 41:1411–1416. doi:10.1007/s10875-021-01027-1

E41. Rae W, Ward D, Mattocks CJ, Gao Y, Pengelly RJ, Patel SV, Ennis S, Faust SN, Williams AP. Autoimmunity/inflammation in a monogenic primary immunodeficiency cohort. *Clin Trans Immunol* (2017) 6:e155. doi:10.1038/cti.2017.38

E42. Lougaris V, Baronio M, Gazzurelli L, Lorenzini T, Fuoti M, Moratto D, Bozzola A, Ricci C, Bondioni MP, Ravelli A, et al. A de novo monoallelic CTLA-4 deletion causing pediatric onset CVID with recurrent autoimmune cytopenias and severe enteropathy. *Clin Immunol* (2018) 197:186–188. doi:10.1016/j.clim.2018.10.007

E43. Le Coz C, Nolan BE, Trofa M, Kamsheh AM, Khokha MK, Lakhani SA, Novelli A, Zackai EH, Sullivan KE, Briuglia S, et al. Cytotoxic T-lymphocyte-associated protein 4 haploinsufficiency-associated Inflammation can occur independently of T-cell hyperproliferation. *Front Immunol* (2018) 9:1715. doi:10.3389/fimmu.2018.01715

E44. Besnard C, Levy E, Aladjidi N, Stolzenberg M-C, Magerus-Chatinet A, Alibeu O, Nitschke P, Blanche S, Hermine O, Jeziorski E, et al. Pediatric-onset Evans syndrome: Heterogeneous presentation and high frequency of monogenic disorders including LRBA and CTLA4 mutations. *Clin Immunol* (2018) 188:52–57. doi:10.1016/j.clim.2017.12.009

E45. Hoyt KJ, Chatila TA, Notarangelo LD, Hazen MM, Janssen E, Henderson LA. The immunologic features of patients with early-onset and polyautoimmunity. *Clin Immunol* (2020) 211:108326. doi:10.1016/j.clim.2019.108326

E46. Crowley E, Warner N, Pan J, Khalouei S, Elkadri A, Fiedler K, Foong J, Turinsky AL, Bronte-Tinkew D, Zhang S, et al. Prevalence and clinical features of inflammatory bowel diseases associated with monogenic variants, identified by whole-exome sequencing in 1000 children at a single center. *Gastroenterology* (2020) 158:2208–2220. doi:10.1053/j.gastro.2020.02.023

E47. Lougaris V, Malagola M, Baronio M, Morello E, Gazzurelli L, Benvenuto A, Palumbo L, Moratto D, Girelli MF, Chiarini M, et al. Successful hematopoietic stem cell transplantation for complete CTLA-4 haploinsufficiency due to a de novo monoallelic 2q33.2-2q33.3 deletion. *Clin Immunol* (2020) 220:108589. doi:10.1016/j.clim.2020.108589

E48. Dalgiç CT, Si̇N AZ, Ardeni̇Z FÖ. Retrospective analysis of autoimmune diseases and immunologic characteristics of the adult primary immune deficiency cohort: 17 years experience of the tertiary referral immunology center in Turkey. *Asthma Allergy Immunol* (2021) 19:12–23.

E49. Krone KA, Winant AJ, Vargas SO, Platt CD, Bartnikas LM, Janssen E, Lillehei C, Lee EY, Fishman MP, Casey A. Pulmonary manifestations of immune dysregulation in CTLA-4 haploinsufficiency and LRBA deficiency. *Pediatr Pulmonol* (2021) 56:2232–2241. doi:10.1002/ppul.25373
